# Development of a STEAP1-Targeted Prostate Cancer Specific Antibody Drug Conjugate Platform with Immunostimulatory Properties

**DOI:** 10.21203/rs.3.rs-9194287/v1

**Published:** 2026-04-24

**Authors:** John Shuhan Wang, Sirajbir S. Sodhi, Li-Chung Tsao, Guang Han Lin, Nicole Moon, Qiang Zhang, Bushangqing Liu, Juliet Penaranda, Timothy N. Trotter, Jason A. Somarelli, Andrew J. Armstrong, H. Kim Lyerly, Guangdi Wang, Zachary C. Hartman

**Affiliations:** Duke University School of Medicine; Duke University School of Medicine; Duke University Department of Surgery; Duke University Graduate School; Duke University; Xavier University of Louisiana; Duke University Graduate School; Duke University; Duke University Department of Surgery; Duke University School of Medicine; Duke University School of Medicine; Duke University Department of Surgery; Xavier University of Louisiana; Duke University Department of Surgery

**Keywords:** Antibody-drug conjugate, STEAP1, androgen pathway modulator resistant disease, immunogenic cell death, Fc-mediated effector function, macrophage antigen presentation, adaptive immune memory, bone metastasis, prostate cancer

## Abstract

**Background:**

Patients with prostate cancer who develop androgen pathway modulation resistant (APMR) disease face lethal outcomes despite advances in androgen receptor–pathway inhibitors, chemotherapy, and PSMA-directed radioligands. Antibody–drug conjugates (ADCs) have demonstrated transformative efficacy in multiple malignancies, yet ADCs evaluated in patients with metastatic APMR have largely focused on direct tumor cytotoxicity without assessing immune mechanisms that may underpin durable therapeutic responses. Whether immune activation contributes to ADC efficacy in prostate cancer remains unexplored.

**Methods:**

We applied immune-centric design principles to ADC development targeting six transmembrane epithelial antigen of the prostate 1 (STEAP1), a prostate-lineage restricted, androgen receptor–regulated surface antigen. vandortuzumab-based ADCs were generated using multiple clinically validated linker–payload platforms. Constructs were evaluated using in vitro assays of Fcγ receptor engagement, antigen presentation, and T-cell activation, followed by in vivo testing in bone-metastatic and syngeneic immunocompetent APMR models.

**Results:**

STEAP1 specific Fcγ receptor engagement was required for optimal antigen presentation and downstream T-cell activation. Among tested payloads, exatecan-based ADCs demonstrated the strongest immunostimulatory activity. In bone-metastatic and immunocompetent APMR models, vandortuzumab–exatecan mediated durable tumor control and induced adaptive immune memory capable of preventing tumor rechallenge.

**Conclusions:**

These findings identify immune activation as an important determinant of ADC efficacy in APMR and demonstrate that vandortuzumab–exatecan functions as an immune-engaging ADC capable of inducing durable anti-tumor responses. Incorporating immune activation into ADC design may improve the therapeutic potential of ADCs for patients with advanced prostate cancer.

## BACKGROUND

Antibody–drug conjugates (ADCs) are targeted cancer therapeutics that combine the specificity of a monoclonal antibody for a tumor-associated antigen with the potent cytotoxicity of a small-molecule payload^1,2^. By binding a specific surface antigen on cancer cells, internalizing, and releasing their cytotoxic payload, ADCs deliver chemotherapy directly to malignant tissue while sparing most normal cells. ADCs have produced durable responses and meaningful survival benefits across multiple malignancies, underscoring their transformative clinical potential^3–5^.

Despite 15 ADC approvals across oncology, highlighted by the pan-solid tumor approval of T-DXd in 2024, no ADCs have been approved for patients with prostate cancer (PC). Six Transmembrane Epithelial Antigen of the Prostate 1 (STEAP1) has nonetheless remained a prime target for multiple APMR-directed ADCs (vandortuzumab vedotin, ABBV-969, and ADRX-0405) and bispecific T cell engager Xaluritamig due to its selective and ubiquitous expression in both primary and metastatic PC^6–10^. Although its precise physiologic role is still being defined, multiple studies show uniform membrane localization and strong expression in > 80% of primary prostate cancers and a high prevalence in bone, lymph-node, and visceral metastases, making it an attractive ADC target^11^. Although prior studies demonstrated adequate localization of STEAP1-targeting ADCs to prostate cancer lesions, early clinical trials have not produced meaningful patient responses. The basis for this limited efficacy remains poorly understood and may reflect incomplete evaluation of the immune effector functions of both the targeting antibody and the ADC constructs derived from it.

In contrast, extensive mechanistic studies of HER2-targeting antibodies and ADCs have revealed that engagement of innate immune pathways can be a critical determinant of therapeutic efficacy^12–14^. For example, our previous studies on combinations of HER2-directed antibodies such as trastuzumab and pertuzumab showed that their anti-tumor activity is closely linked to macrophage-mediated antibody-dependent cellular phagocytosis (ADCP) and enhanced complement deposition on tumor cells, which increases opsonization and promotes phagocytic clearance^12^. Moreover, our recent comparative mechanistic analyses of trastuzumab deruxtecan (T-DXd) and its predecessor trastuzumab emtansine (T-DM1) demonstrated that coordinated engagement of innate and adaptive immunity distinguishes these agents and likely contributes to durable therapeutic responses^15^. We found that the topoisomerase inhibitor (TOPOi) DXd elicited higher levels of immunogenic cell death (ICD) compared with microtubule inhibitor DM1, and that tandem Fc engagement and ICD synergize to activate innate immune responses that promote the expansion of tumor antigen-specific adaptive immunity. This immune activation ultimately underlies the superior anti-tumor activity of T-DXd in HER+ breast cancer patients, a finding that is mirrored by improved clinical outcomes from T-DXd treatment in comparison to T-DM1^15,16^.

Nevertheless, immune mechanisms including Fc receptor engagement, complement activation, macrophage-mediated phagocytosis, and payload-driven immunogenicity have not been systematically evaluated in the development of antibodies or ADCs targeting STEAP1 in APMR^8,17,18^. Whether similar biology governs the activity of STEAP1-directed ADCs in prostate cancer remains unknown. Progress in this area has also been limited by the scarcity of clinically relevant syngeneic prostate cancer models capable of tolerating xeno-antigen expression in immunocompetent hosts while maintaining therapeutic responsiveness^19–23^.

In this study, we engineered a series of antibody–drug conjugates (ADCs) derived from the anti-STEAP1 antibody vandortuzumab and performed comparative functional analyses of their ability to activate innate immune pathways and promote tumor-specific T-cell responses. We then evaluated the therapeutic potency and capacity of the lead candidate, vandortuzumab–exatecan, to induce durable adaptive immune memory in a newly developed syngeneic immunocompetent prostate cancer model. In parallel, we confirmed its anti-tumor activity across multiple human prostate cancer xenograft models in immunodeficient mice, including a bone-metastatic model that recapitulates the predominant metastatic niche of prostate cancer.

## METHODS

### Study Approval

All animals were maintained and bred in accordance with Duke Institutional Animal Care and Use Committee–approved protocol (A043-23-02) and supervised by Division of Laboratory Animal Resources (DLAR).

### Therapeutic antibodies, antibody-drug-conjugates, and unconjugated payloads

Vandortuzumab (anti-STEAP1 IgG1) and A1 were cloned into pcDNA3.4 TOPO vectors and expressed after transfection into Expi293F cells. Antibodies were then purified from supernatants by Protein G affinity chromatography. Fc-silent variants (LALAPG) were generated via site-directed mutagenesis and verified by sequencing. ADCs were generated using standard cysteine-based conjugation chemistry following partial interchain disulfide reduction. Vandortuzumab was conjugated to VC–MMAE, VC–exatecan, or GGFG–DXd linkers as described previously^24^. Vandortuzumab ADC constructs were characterized by high-resolution mass spectrometry using an Orbitrap instrument and reverse-phase high-performance liquid chromatography (RP-HPLC). ADC samples were denatured prior to analysis to enable resolution of individual light and heavy chains.

Mass spectra were acquired in the m/z range of 2,000–3,500 to detect multiply charged ions, and deconvolution was performed to determine the molecular masses of conjugated species and calculate the average drug-to-antibody ratio (DAR). All ADC preparations were dialyzed against lyophilizing buffer, sterile-filtered and stored at 4°C until use^25^. Unconjugated payloads were obtained commercially from MCE (#HY13631E, HY-147095, HY-145929, HY-153069, HY-15575) and diluted in DMSO.

### Cell lines and genetic modifications strategies

Human prostate cancer cell lines 22Rv1, PC3, and C4-2B and murine EMT-6 cells were obtained from ATCC. Murine PT-09 cells, derived from PTEN/TP53-deficient prostate tumors on a C57BL/6 background, were kindly provided by Dr. Brian Ruffell at Moffitt Cancer Center and have been previously characterized^26^. Human cell lines were maintained in RPMI-1640, and murine cell lines were maintained in DMEM, each supplemented with 10% fetal bovine serum and 1% penicillin–streptomycin. All cells were cultured at 37°C in 5% CO_2_.

Stable expression of human STEAP1 was achieved via lentiviral transduction. Human cell lines were transduced using a pLenti-CMV-Puro vector, whereas murine PT-09 and EMT-6 cells were transduced using a pCDH-CMV expression vector. For selected experiments, 22Rv1 were additionally engineered to express membrane-bound ovalbumin (mOVA) or eGFP-Luciferase under pLenti-CMV-Hygro and pCDH vector, respectively. Transduced populations were sorted by flow cytometry to establish stable expressing lines.

STEAP1 knockout cell lines were generated using CRISPR/Cas9-mediated gene editing. Guide RNAs targeting exon 2 of STEAP1 (gRNA: TTTAGAAGAAGACGATTATT) were cloned into pLentiCRISPRv2, packaged into lentivirus, and used for transduction. Following antibiotic selection, knockout populations were enriched and sorted by flow cytometry prior to validation.

### In vitro ADC and payload cytotoxicity assays

22Rv1.hSTEAP1, PC-3.hSTEAP1, EMT-6.hSTEAP1, and PT-09.hSTEAP1 were incubated in 96-well culture plate (1–5000 cells/well) containing titrated ADCs, antibodies, or unconjugated payloads at equimolar concentration to ADCs. Treated cells were cultured for 4 days, and cell viability was assessed by total cellular ATP quantification using CellTiter-Glo Luminescent Cell Viability Assay (Promega, #G7571).

### ADC internalization and endocytosis assessment

One mg of Ab or ADC were labeled with pHrodo using Deep Red pHrodo Antibody Labeling Kit (Thermo, P35356) following manufacturer’s protocol. Labeled ADCs were added to 22Rv1.hSTEAP1 and PC-3.hSTEAP1. 24 h after incubation, cells were harvested and analyzed by flow cytometry for internalization. In parallel, cells were seeded onto MATTEK glass-bottom dishes (P12G-0-10-F), treated under identical conditions, and imaged using a CellDiscoverer 7 microscope.

### Bystander killing analysis

22Rv1.hSTEAP1 and 22Rv1.Parental cells were labeled with CellTrace Far Red and CellTrace Violet, respectively according to manufacturer’s protocol. Labeled cells were washed twice with PBS, and co-cultured together at a 4:1 ratio of STEAP1-positive cells versus STEAP1-negative cells (200,000 total cells in 12-well plates). Cells were treated with ADCs (1 ug/mL), and apoptosis of 22Rv1 cells were analyzed on day 6 post-treatment by Zombie-NIR live dead staining (Biolegend, 423105).

### Cathepsin L and B activity assay and assessment

All reagents were purchased from R&D System. Recombinant human CTSL (#952-CY) and CTSB (#953-CY) were dissolved in assay buffers specific to their pH requirement. CTSL and CTSB assay buffer: 50 mM MES, 5 mM DTT, 1 mM EDTA, and 0.005% Brij-35, pH 6.0. A fluorogenic peptide substrate, Z-Leu-Arg-AMC (#ES008), was used as previously described to measure cleavage activity on fluorescent plate reader (Ex/Em 380/460 nm)^15^. For cleavage of STEAP1-ADCs, CTSL or CTSB (1 μg/mL) were incubated with 2 mg/mL of ADCs for 6 h. Cleaved ADCs were added to 22Rv1.Parental cells to access cytotoxicity of released payloads.

### Prostate cancer biospecimen and tissue microarray analysis

Prostate Cancer TMA was purchased from the Cooperative Human Tissue Network (CHTN) (https://chtn.sites.virginia.edu/chtn-prcprog1). TMAs were assessed for STEAP1, CTSB, and CTSL expression via IHC analysis using STEAP1 antibody EPR29701-75 (1:250 dilution), CTSB antibody clone D1C7Y (1:1000 dilution), CTSL antibody clone E3R3P (1:1000 dilution). High-resolution images were acquired using the Cytation7 machine. QuPath software was used for Pixelwise quantification of CTSL DAB staining intensity and H-score calculation following a previously published method^27^.

### FCGR activation assay

JURKAT cells expressing human FCGR3A or mouse FCGR4 with NFAT-Luciferase reporter were generated with lentiviral transduction and selected with puromycin, as validated in our previous publications^13^. PC-3.hSTEAP1 cells were first plated and treated with serially diluted doses of isotype, vandortuzumab, A1, or ADCs for 30 minutes. Jurkat-FCGR-NFAT-LUC effector cells were added and co-cultured for 18 hours. FCGR signaling activation was assessed by luciferase activity quantification.

### Primary mouse macrophages generation

Mouse BMDMs were generated from isolated bone marrows in the mouse femur and tibia, differentiated for 7–8 days with L929 conditioned media (LCM). A total of 50 million bone marrow cells were plated in 10 cm^2^ dishes with 20% LCM on day 0. Unattached cells in the supernatant were removed, and fresh media was supplemented on days 3, 5, and 7.

### In vitro ADCP assays

BMDMs were used for ADCP assays on day 7 of differentiation. 22Rv1.hSTEAP1 and PC-3.hSTEAP1 were labeled with pHrodo Green AM (ThermoFisher, P35373). CTV-labeled BMDMs (50,000 cells) and labeled target cells (100,000 cells) were incubated with (1 μg/mL) Ab or ADC in 96-well plates for 4.5 hours at 37°C. After co-culture, phagocytosis of labeled tumor cells by macrophages was analyzed by flow cytometry as per previously published methods^28^.

### In vitro complement assays

C4-2B, 22Rv1.hSTEAP1 parental/ CM, and PC-3.hSTEAP1 were incubated with 1 μg/mL Ab or ADC for 30 minutes at 37°C. After incubation, freshly isolated mouse serum from SCID-Beige or C1q−/− SCID-Beige mice were added to culture to a final concentration of 10% serum. Cells were cultured at 37°C for another 1 hour, washed once with PBS, and stained with anti–mouse C3 antibody (Abcam, 12E2) and FACS analysis of live C3b+ cells percentage.

PC3.hSTEAP1 was incubated with titrations of Ab or ADC for 30 minutes at 37°C. After incubation, rabbit serum (non-heat-inactivated) were added to culture to a final concentration of 10% serum. After 4–6 hours, complement dependent cytotoxicity was assessed using a CellTiter-Glo luminescent assay.

### In vitro macrophage activation analysis by flow cytometry and RNA-Seq

22Rv1.hSTEAP1 CM and PC-3.hSTEAP1 (500,000 cells) were pre-treated with 1 μg/mL of Ab, ADC, or DAR equivalent unconjugated payloads for 30 minutes. C57Bl/6 BMDMs (250,000 cells) were then added and co-cultured for 2 days. On the day of analysis, macrophages/DCs were harvested using a cell scraper after 30 min incubation with Accutase. Antigen-presentation and activation surface markers were analyzed by flow cytometry. FcR blockers (Biolegend, 101319) were used before staining with flow antibodies. The following mouse panel was used: CD40 PE, CD80 BV650, MHC-II BV785, MHC-I Alexa Fluor 647, CD11b PE-Fire 640, CD86 PE-Cy7. Representative FACS gating strategies can be found in Supplementary Fig. 4B. Activated macrophages from a repeat two-day co-culture experiment with 22Rv1.hSTEAP1 CM were used for RNA-Seq analysis. Total RNA was harvested using RNeasy plus mini kit (Qiagen, 74134). RNA quality and concentration were determined using the Qubit RNA HS Assay Kit (Q32852). Transcript quantification was performed using Salmon (v1.x) with quasi-mapping against a combined mouse and human reference transcriptome (GRCm38/mm10 and GRCh38/hg38)^29^. Transcript-level abundance estimates were imported into R (v4.3.0) using tximport to generate gene-level count matrices for downstream analysis^30^. Only reads mapping to the mouse transcriptome were retained for downstream analysis, and human-derived transcripts were excluded. Differential expression analysis was performed using DESeq2^31^. Low-abundance genes were filtered prior to analysis, and size-factor normalization was applied using the DESeq2 median-of-ratios method. Differentially expressed genes (DEGs) were identified using the DESeq2 negative binomial generalized linear model with default parameters. For visualization and clustering analyses, normalized counts were transformed using the variance-stabilizing transformation (VST). DEGs were defined using an adjusted p-value (Benjamini–Hochberg correction) < 0.05 and an absolute log_2_ fold-change ≥ 1 unless otherwise specified. Gene set enrichment analysis and pathway analysis were performed using clusterProfiler and ReactomePA packages in R^32,33^. Gene set enrichment analysis was additionally performed using the fgsea R package (v1.x) against the MSigDB Hallmark gene collection (v2023.1), accessed via the msigdbr R package^34^. Genes were pre-ranked by DESeq2 Wald statistic. Gene sets with fewer than 15 or more than 500 genes were excluded. Normalized enrichment scores (NES) and adjusted p-values were calculated using 1,000 permutations, with significance defined as adjusted p < 0.05.

### OTI T cell co-culture and activation

22Rv1.hSTEAP1 CM cells were further engineered to express membrane-bound ovalbumin (mOVA) via lentiviral transduction followed by hygromycin selection. For co-culture experiments, 2 × 10^5^ tumor cells were treated with antibody, ADC, or DAR-equivalent unconjugated payload for 30 minutes. Subsequently, 1 × 10^5^ C57BL/6 BMDMs were added and co-cultured with tumor cells for 48 hours to allow phagocytosis and macrophage activation.

After co-culture, tumor cells were selectively removed by brief trypsinization. Macrophages were washed and maintained in fresh T-cell media (RPMI supplemented with 50 μM 2-mercaptoethanol and 100 IU/mL murine IL-2). Whole splenocytes were isolated from OT-I transgenic mice, and CD8^+^ T cells were purified (STEMCELL Technologies, 19853), labeled with CellTrace Violet, and added to macrophage cultures at 1 × 10^5^ cells per well. As controls for T-cell activation, SIINFEKL peptide or mock eGFP peptide (1 μg/mL) was included in parallel wells.

After 72 hours of co-culture, T cells were harvested and analyzed by flow cytometry for proliferation and activation using antibodies against CD8 (APC) and CD44 (PerCP-Cy5.5), along with ViaDye Red for viability assessment. Representative gating strategies are shown in Supplementary Fig. 4J, and peptide control histograms are provided in Supplementary Fig. 4K.

### Mouse strains

All mouse experiments were performed on male mice for relevance in prostate cancer studies. SCID-beige (C.B-*Igh*-1b/GbmsTac-*Prkdc^scid^-Lyst^bg^* N7; Taconic Biosciences) mice between the ages of 6 and 10 weeks old were used for all human cell line xenograft experiments. BALB/c (#BALB/cAnNTac) and C57Bl/6J (#000664) were purchased from Taconic and the Jackson Laboratory, respectively. All experiments were performed with a minimum of n = 5 per treatment group, except for isotype control in PC3.hSTEAP1 in SCID-Beige (n = 4).

### Subcutaneous implanted xenograft models and therapeutic ADC/antibody treatments

22Rv1.hSTEAP1 CM and PC-3.hSTEAP1 cells (5 × 10^5^) were suspended in 50% Matrigel/PBS and subcutaneously implanted into SCID-beige mice. Tumor growth was monitored over time using caliper measurements, and tumor volume was calculated using the formula (length × width^2^)/2. For therapeutic studies, mice bearing established PC-3.hSTEAP1 tumors were randomized and treated with a single intraperitoneal dose (4 mg/kg) of isotype control antibody, parental vandortuzumab, or vandortuzumab–exatecan at the indicated time points post-implantation. Mice bearing 22Rv1.hSTEAP1 CM tumors were randomized and treated with two intraperitoneal doses (2 mg/kg) of isotype antibody, vandortuzumab, or vandortuzumab-exatecan administered three days apart.

### Bone-metastatic xenograft models and therapeutic ADC/antibody treatments

22Rv1.hSTEAP1.eGFP-Luciferase cells (1 × 10^6^ in PBS) were injected into the caudal artery of SCID-Beige mice. Mice were monitored weekly by bioluminescence imaging until tumor signal was detected. For imaging, mice were administered 50 uL of D-luciferin (30 mg/kg, intraperitoneal), and images were acquired 10 minutes post-injection using an IVIS XR system. Acquisition parameters were as follows: open emission filter, exposure time 60 s, medium binning (8), and f/stop 1. Bioluminescent images were analyzed using Living Image 4.3 software (PerkinElmer). Mice bearing established 22Rv1.hSTEAP1 CM bone metastases were randomized and treated with four intraperitoneal doses (4 mg/kg) of isotype control antibody or vandortuzumab–exatecan administered weekly.

### Syngeneic PT-09 Model in C57Bl/6

PT-09 cell line were kindly provided by Brian Ruffell at Moffitt Cancer Center under a material transfer agreement with J.S.. 4 x 10^5^ tumor cells in 1:1 ratio of PBS and matrigel were implanted on the right flank. Tumors were measured twice a week as previously described. Mice were treated with five doses of isotype antibody or vandortuzumab–exatecan administered every three days. Animals exhibiting complete tumor regression were maintained for two additional weeks with continued twice-weekly monitoring to assess tumor relapse prior to rechallenge. Mice were then rechallenged with 4 × 10^5^ PT-09.hSTEAP1 and PT-09 parental cells implanted on the bilateral flanks. Control animals received freeze–thaw–treated PT-09.hSTEAP1 and PT-09 parental cells on the corresponding flanks two weeks prior to rechallenge. Tumor growth following rechallenge was monitored for 4 weeks, with measurements performed twice weekly.

### Flow Cytometry analysis of tumor-infiltrating immune cells

When EMT6.hSTEAP1 reached humane endpoint size (> 500–1000 mm^3^), whole tumors from mice were harvested and chopped into < 1 mm pieces and incubated for 1 hour in digestion buffer (DMEM + 100 μg/mL collagenase + 0.2 U/mL DNAse + 1 μg/mL hyaluronidase). Single-cell suspensions were spun down through a 40 μm filter and washed with media. Approximately 1–2 million cells were used for staining and flow cytometry analysis on Cytek Northern Lights machine. Cells were treated with FcR blocker for 15 minutes prior to staining with flow antibodies.

The following panel of immune cell markers (BioLegend, unless otherwise specified) were used: viability dye Zombie NIR, CD45 APC-Fire 810, CD3 APC-Fire 750, CD4 BV750, CD8 SB645, CD69 Spark Blue 550, CD44 SB600 (eBioscience), CD19 BV510, CD49b PE-Cy7, CD11b BV570, Ly6G Spark NIR 685, Ly6C PE-Cy5, F4/80 eFluor 450 (eBioscience), and CD11c AF700. Tumor-infiltrating T cells were identified by CD8 and CD4 gating in the live CD45+/CD11b−/CD3 + population. CD44 MFI was used to identify activated CD8 T cells. Macrophages were identified as CD45+/CD11b+/F4/80+/Ly6G-population. Representative FACS gating strategies can be found in Supplementary Fig. 5C.

### Immunohistochemistry analysis of tumor

PT-09.hSTEAP1 tumors were fixed in 10% formalin for 48 hours, processed, and embedded in paraffin. Sectioned tumors were then stained with anti-mouse CD8 (1:500, Cell Signaling Technology, #D4W2Z) or F4/80 (1:500, Cell Signaling Technology, #D2S9R) antibodies with hematoxylin counterstaining. The number of CD8 + cells or % area of F4/80 stain per 20x field, averaged across five fields per replicate, was quantified through Fiji/ImageJ.

### Public Transcriptomic Dataset Analysis (TCGA, GTEx, and SU2C)

Publicly available transcriptomic datasets were used to evaluate STEAP1, CTSL, and CTSB expression across primary and advanced prostate cancer samples. RNA-sequencing data from The Cancer Genome Atlas (TCGA) Prostate Adenocarcinoma (PRAD) cohort and corresponding normal prostate tissue data from the Genotype-Tissue Expression (GTEx) project were accessed through the UCSC Xena Browser. STEAP1 Gene expression values were obtained as log_2_-transformed transcripts per million [log₂(TPM + 1)]. Tumor and normal samples were compared to assess differential expression across disease states.

mAPMR transcriptomic data were obtained from the SU2C/PCF Dream Team cohort comprising metastatic biopsy samples from patients with advanced prostate cancer^35^. STEAP1, CTSL, and CTSB expression data were downloaded from cBioPortal. Gene expression values were analyzed as log_2_(TPM + 1). Comparisons across metastatic sites were performed without site-specific normalization beyond that provided in the processed dataset. All data processing and visualization were performed in R (v4.3.0).

### Statistical analysis

Statistics were performed using Prism 9 (GraphPad Software). All data were assumed a normal distribution. One-way analysis of variance (ANOVA) tests with Tukey’s multiple comparisons tests were performed to determine differences in multi-groups experiments. Log-rank (Mantel–Cox) test was used for animal survival analysis. P < 0.05 was considered statistically significant. All data plotted are presented as mean ± SD.

## RESULTS

### Validation of anti-STEAP1 antibodies and characterization of innate immune activation

**Figure 1:**
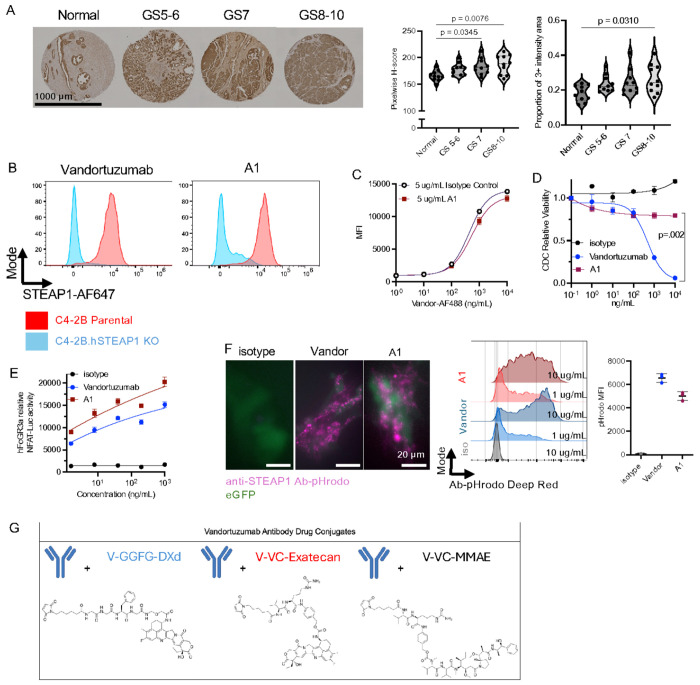
STEAP1 expression in prostate cancer and characterization of anti-STEAP1 antibodies. (A) Representative immunohistochemistry (IHC) images of STEAP1 protein expression in normal prostate epithelium and primary prostate adenocarcinomas stratified by Gleason score (5–6, 7, and 8–10). Plasma-membrane staining was quantified by H-score (product of staining intensity and percentage of cells at each intensity level). Scale bars, 1000 μm. (B) Binding affinity of anti-STEAP1 antibodies A1 and vandortuzumab measured by flow cytometry on STEAP1-positive and negative C4-2B. (C) Competitive binding assay on STEAP1+ NIH-3T3 cells. Vandortuzumab-AF488 was titrated in the presence of saturating concentrations of unlabeled A1 (5 μg/mL). Rightward shift of the IC_50_ (1,006 ng/mL without competitor vs. 3,109 ng/mL with A1). (D) Complement-dependent cytotoxicity (CDC) of titrated vandortuzumab versus A1 and isotype control on PC3.hSTEAP1 cells in the presence of 10% rabbit complement. (E) FcγRIIIa activation assessed by NFAT-Luciferase reporter assay in JURKAT cells expressing human FcγRIIIa co-cultured with PC3.hSTEAP1 cells treated with vandortuzumab, A1, or isotype control. (F) Internalization of pHrodo-labeled isotype antibody, vandortuzumab and A1 assessed by flow cytometry and fluorescence microscopy in 22Rv1.hSTEAP1.eGFP-Luc cells at 24 hours. Increased pHrodo fluorescence indicates endosomal acidification consistent with receptor-mediated internalization. Scale bar, 20 μm. (G) Schematic of the ADCs evaluated in this study, including vandortuzumab–VC-MMAE, vandortuzumab–VC-exatecan, and vandortuzumab–GGFG-DXd. All conjugates exhibited average drug-to-antibody ratios (DARs) of 7–8. Data are represented as mean ± SD. Statistical comparisons by one-way ANOVA with Tukey’s multiple comparisons test. Dose–response curves were fit using a four-parameter logistic (4PL) regression model.

We first assessed STEAP1 protein expression across increasing grades of prostate cancer using immunohistochemistry (IHC). Tissue microarrays included normal prostate epithelium and primary adenocarcinomas stratified by Gleason score (5–6, 7, and 8–10). Plasma-membrane STEAP1 staining was quantified by using an H-score as the product of staining intensity (0–3) and the percentage of tumor cells at each intensity level. High-grade tumors (Gleason 8–10) showed significantly greater STEAP1 expression (Fig. 1A, Supplementary Fig. 1A), including a larger fraction of tumor cells exhibiting strong (3+) membrane staining. These findings were consistent with independent STEAP1 mRNA expression data from the TCGA–GTEx cohort (Supplementary Fig. 1B). Further, we evaluated STEAP1 mRNA expression across different metastatic sites in the SU2C-PCF mAPMR cohort^36^. STEAP1 expression varied significantly across metastatic tissue sites, where bone and lymph node (LN) metastases exhibited the highest STEAP1 mRNA expression levels (log2(TPM + 1), while liver metastases showed comparatively lower and more variable expression (Supplementary Fig. 1C).

Multiple antibodies targeting STEAP1 have been previously developed, and we next compared the binding affinity and specificity of two candidates: A1, a converted IgG1 of the parental bispecific Xaluritamig by Amgen, and vandortuzumab, the parental antibody designed by Agensys, Inc. used in the clinically developed conjugate vandortuzumab vedotin^10,37^. Both A1 and vandortuzumab displayed similar high affinity for STEAP1 (Fig. 1B, Supplementary Fig. 1D). A1 showed slightly reduced specificity, evidenced by higher binding to C4-2B STEAP1-knockout cells. To determine whether these antibodies recognize distinct STEAP1 epitopes, we performed a competitive binding assay using NIH-3T3 engineered to express human STEAP1. Vandortuzumab was labeled with Alexa Fluor 488 (AF488) and titrated in the presence of saturating concentrations of unlabeled A1 (5 μg mL^−1^). Increasing concentrations of vandortuzumab-AF488 demonstrated moderate competition between the two antibodies, with an IC_50_ of 1,006 ng mL^−1^ for vandortuzumab alone and 3,109 ng mL^−1^ in the presence of A1, while achieving similar maximum mean fluorescence intensities (Fig. 1C). Indeed, our experimental model were in accordance with prior cryo-EM findings that A1 and vandortuzumab occupy partially overlapping binding sites^10,38^.

We next evaluated whether the two antibodies exerted direct anti-proliferative effects on STEAP1-positive APMR cell lines, 22Rv1 and C4-2B. Neither antibody inhibited tumor cell growth in vitro (Supplementary Fig. 1E). In contrast to antibodies or ADCs whose target engagement can directly impair tumor cell proliferation, these findings suggested that anti-STEAP1 antibodies are unlikely to mediate substantial tumor control through direct growth inhibition alone^39^. We therefore reasoned that their therapeutic activity, particularly when conjugated to cytotoxic payloads, would depend on Fc-mediated effector mechanisms, including complement activation and Fcγ receptor (FcγR) engagement. These mechanisms, as demonstrated for HER2-directed antibodies and ADCs, promote complement opsonization of tumor cells and allows for macrophage-mediated ADCP^12^.

To evaluate classical complement activation and complement-dependent cytotoxicity (CDC), PC3 STEAP1^+^ cells were incubated with titrated concentrations of A1 or vandortuzumab for 30 minutes, followed by addition of 10% (v/v) rabbit complement. Cells were then incubated for 1 hour, and cytotoxicity was quantified using CellTiter-Glo. Despite similar binding characteristics, vandortuzumab robustly triggered complement activation, whereas A1 did not (Fig. 1D). Using IgG-depleted human complement and measuring C3b/iC3b deposition on the cell surface, we further confirmed that vandortuzumab induced strong C3 deposition, while A1 showed minimal activity, consistent with our CDC results (Supplementary Fig. 1F). We next assessed Fcγ receptor engagement using our previously described FcγRIIIa JURKAT reporter assay, which models the predominant Fcγ receptor mediating antibody-dependent cellular phagocytosis (ADCP)^13^. Both antibodies induced greater FcγRIIIa activation than the isotype control (Fig. 1E). Consistent with these findings, activation of murine FcγRIV, the functional homolog of human FcγRIIIa, showed a similar pattern in JURKAT reporter assays (Supplementary Fig. 1G). Finally, we confirmed that both antibodies were efficiently internalized by tumor cells, supporting their potential utility as antibody–drug conjugates (Fig. 1F, Supplementary Fig. 1H). Based on its robust complement activation, conserved ability to elicit FcγR signaling, and established clinical track record as an IgG1 antibody, we selected vandortuzumab as the template for further development^6^.

### Development of Vandortuzumab Antibody Drug Conjugates and In Vitro Cytotoxicity

**Figure 2:**
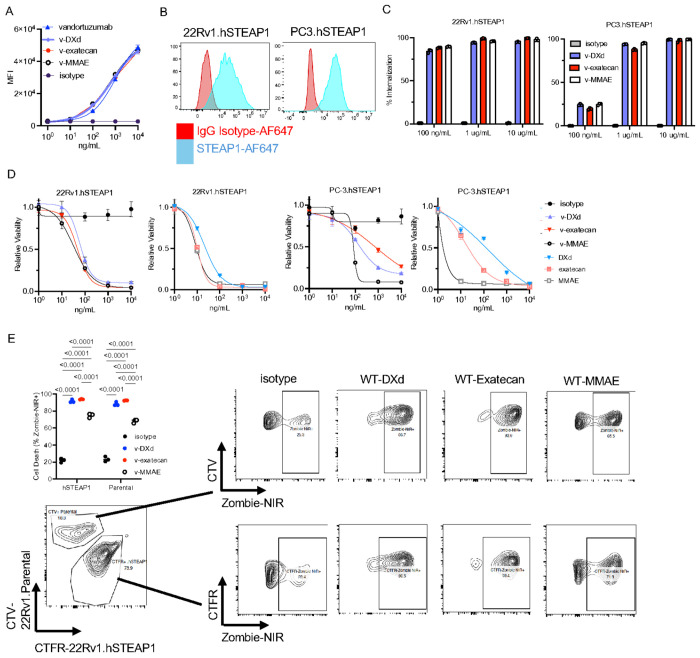
Development and in vitro characterization of vandortuzumab antibody–drug conjugates. (A) STEAP1 binding of vandortuzumab–VC-MMAE, vandortuzumab–VC-exatecan, and vandortuzumab–GGFG-DXd compared to parental vandortuzumab by titration flow cytometry on STEAP1-positive cells. (B) STEAP1 surface expression on 22Rv1.hSTEAP1 and PC3.hSTEAP1 cell lines by flow cytometry. (C) Antigen-dependent internalization of pHrodo-labeled ADCs in 22Rv1.hSTEAP1 and PC3.hSTEAP1 cells at 24 hours assessed by flow cytometry. (D) In vitro cytotoxicity dose-response curves (IC_50_) for vandortuzumab–MMAE, vandortuzumab–exatecan, vandortuzumab–DXd, and equimolar unconjugated payloads in 22Rv1.hSTEAP1 and PC3.hSTEAP1 cells after 96-hour co-culture. Cell viability was assessed by CellTiter-Glo. (E) Bystander killing assay. CellTrace Far Red–labeled 22Rv1.hSTEAP1 cells and CellTrace Violet–labeled 22Rv1 parental cells were co-cultured at a 4:1 ratio and treated with ADCs (1 μg/mL) for 6 days. Apoptosis in each population was assessed by Zombie-NIR live/dead staining. Data are represented as mean ± SD. Statistical comparisons by one-way ANOVA with Tukey’s multiple comparisons test. Dose–response curves were fit using a 4PL regression model.

We next conjugated vandortuzumab to VC–exatecan, VC–MMAE, and GGFG–DXd using standard cysteine-based linker chemistry, achieving average drug-to-antibody ratios (DARs) of 7–8 (Fig. 1G, Supplementary Fig. 2A–F)^24^. VC–MMAE was included as a benchmark, as it represents the clinically evaluated vandortuzumab vedotin construct. In parallel, we incorporated two topoisomerase I–based payloads, exatecan and deruxtecan. VC–exatecan was selected to maintain a consistent valine–citrulline linker backbone, enabling direct comparison of payload-specific effects between MMAE and exatecan. GGFG–DXd was included given the clinical success of this linker–payload platform in HER2-directed ADCs. All conjugates retained STEAP1 binding comparable to the parental antibody as demonstrated by titration analysis via flow cytometry (Fig. 2A).

For in vitro studies, we employed 22Rv1 and PC3 cells transduced to express human STEAP1 (Fig. 2B). All ADC constructs demonstrated antigen-dependent internalization in both cell lines (Fig. 2C). Internalization approached near-complete uptake at higher antibody concentrations, confirming that STEAP1 is capable of efficient endocytic trafficking. At lower concentrations (100 ng/mL), however, PC3.hSTEAP1 cells exhibited reduced internalization relative to 22Rv1.hSTEAP1, suggesting cell line–specific differences in antigen density, trafficking kinetics, or antibody engagement. We next assessed direct cytotoxicity (Fig. 2D). In 22Rv1.hSTEAP1 cells, all ADCs exhibited comparable IC_50_ values. In contrast, in PC3.hSTEAP1 cells, WT–MMAE was the most potent construct, followed by WT–DXd and WT–exatecan. Payload-only controls were tested at equimolar concentrations matched to the effective DAR of each ADC. Across both models, ADCs demonstrate weaker potency than their corresponding free payloads. Given the confirmed capacity for ADC internalization, this finding likely reflects differences in the proportion of antigen engaged and internalized at therapeutically relevant concentrations, which may limit intracellular payload accumulation relative to more highly expressed or rapidly cycling targets.

We next evaluated the capacity of each ADC to induce bystander killing. ADC-sensitive 22Rv1.hSTEAP1 cells were co-cultured with antigen-low, ADC-insensitive parental 22Rv1 cells at a 4:1 ratio for 5 days. Under these conditions, vandortuzumab–DXd and vandortuzumab–exatecan mediated near-complete elimination of parental cells, consistent with effective bystander activity. In contrast, vandortuzumab–MMAE demonstrated reduced bystander killing. Notably, cell death within the 22Rv1.hSTEAP1 population was also lower in the vandortuzumab–MMAE condition at this later time point, potentially reflective of the more rapid cytotoxic kinetics of the vandortuzumab–MMAE construct or selection of pre-existing resistant cells during the extended co-culture period (Fig. 2E).

### Differential Susceptibility of ADC Linkers to Cathepsin B and L

**Figure 3:**
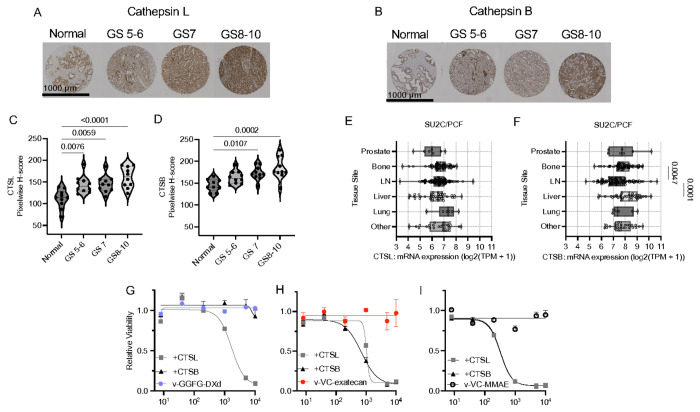
Cathepsin B and L expression in prostate cancer and differential ADC linker cleavage. (A–B) Representative IHC images of Cathepsin L (CTSL) and Cathepsin B (CTSB) expression in normal prostate epithelium and primary prostate adenocarcinoma by Gleason grade categories on a tissue microarray (TMA). (C-D) H-score quantification of CTSL and CTSB expression across normal and malignant prostate specimens on TMA. (E-F) CTSL and CTSB mRNA expression across metastatic biopsy sites (bone, lymph node, liver, lung) in the SU2C/PCF mAPMR cohort, visualized as log_2_(TPM + 1). (G) Cytotoxicity rescue assay demonstrating cleavage of vandortuzumab–GGFG-DXd by recombinant CTSL but not CTSB. Cleaved ADC conditioned media was added to STEAP1-low 22Rv1 parental cells for 72 hours and viability was measured by CellTiter-Glo. (H) Cytotoxicity rescue assay demonstrating cleavage of vandortuzumab–VC-exatecan by both recombinant CTSL and CTSB. (I) Cytotoxicity rescue assay for vandortuzumab–VC-MMAE, demonstrating cleavage by both CTSL and CTSB. H-scores are shown as median with interquartile range; transcript levels are shown as median with min–max. Statistical comparisons were performed using one-way ANOVA with Tukey’s multiple comparisons test. Dose–response curves were fit using a 4PL regression model.

Given that ADC linker cleavage is mediated by different lysosomal proteases, we first assessed expression of Cathepsin L (CTSL) and Cathepsin B (CTSB) in our prostate cancer tissue microarray to define the proteolytic landscape of the disease^40,41^. IHC staining revealed a clear increase in CTSL and CTSB expression in prostate adenocarcinoma compared with normal prostate epithelium (Figs. 3A–B, Supplementary Figs. 3A–B). Elevated expression was maintained across Gleason score categories without a statistically significant stepwise increase with grade (Figs. 3C–D), indicating that upregulation of these proteases is an early and sustained feature of malignant transformation rather than one that progressively escalates with tumor grade. We next interrogated transcriptomic data from the SU2C/PCF Dream Team cohort, comprising 444 patients with APMR, to evaluate CTSL and CTSB expression across disease sites (Fig. 3E–F)^36^. Gene expression values were analyzed as log_2_(TPM + 1). Although primary tumor biopsies were limited in this cohort, both CTSL and CTSB expression were consistently maintained across metastatic sites, without evidence of site-specific loss. These findings suggest that expression of these lysosomal proteases is preserved in advanced APMR, including metastatic disease.

To directly evaluate protease-dependent cleavage, we utilized parental 22Rv1 cells, which express low STEAP1 and were found to be insensitive to our ADC constructs. This model allowed us to assess whether exogenous addition of CTSL or CTSB, and consequent linker cleavage, would restore cytotoxic sensitivity independent of antigen-mediated internalization. These assays revealed that vandortuzumab–DXd, which employs a GGFG linker, was cleaved by CTSL but not CTSB (Fig. 3G). In contrast, vandortuzumab–exatecan and vandortuzumab–MMAE, both incorporating a valine–citrulline linker, were cleaved by both CTSL and CTSB (Figs. 3H and 3I). To determine whether linker composition alone dictated protease susceptibility, we further generated a GGFG-linked exatecan construct incorporating a PABC spacer (vandortuzumab–GGFG–exatecan). This construct surprisingly demonstrated sensitivity to both proteases, although cleavage by CTSB was less efficient than by CTSL (Supplementary Fig. 3C). Collectively, these results indicate that protease susceptibility is not determined solely by linker sequence but is also influenced by the structural context and spatial positioning of the payload relative to the linker.

### Vandortuzumab ADCs retain parental FcγR signaling activity and Complement Activation

**Figure 4:**
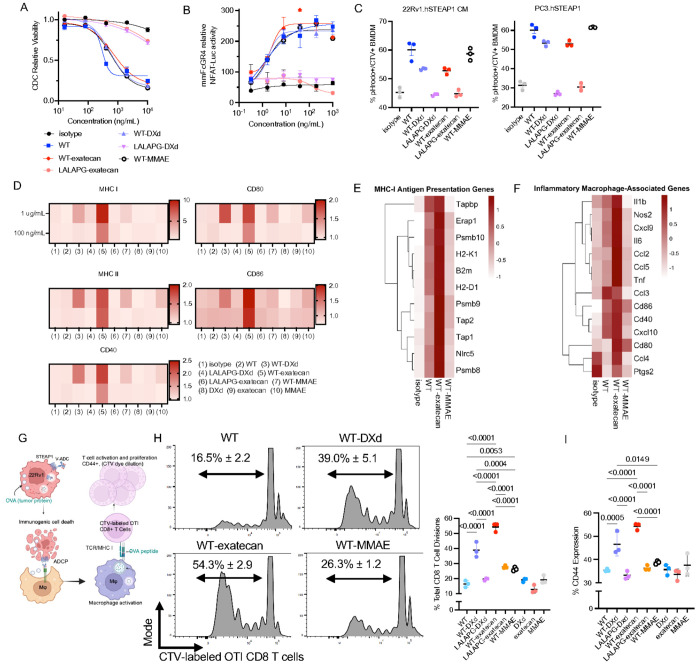
Fc engagement and topoisomerase-I payloads cooperate to drive innate immune activation and T-cell priming. (A) Classical complement pathway activation by wild-type (WT) versus LALAPG Fc-silent vandortuzumab ADCs after 1 hour incubation of PC3.hSTEAP1 cells with 10% v/v rabbit complement, assessed through CellTiter Glo. (B) FcγR signaling capacity of vandortuzumab ADCs assessed by NFAT-Luciferase Jurkat reporter assays for murine FcγRIV. (C) Macrophage-mediated ADCP of pHrodo Green AM-labeled PC3.hSTEAP1 and 22Rv1.hSTEAP1 CM cells by CellTrace Violet–labeled murine bone marrow–derived macrophages (BMDMs) after 4-hour co-culture. (D) Macrophage activation surface markers (MHC I, MHC II, CD40, CD80, CD86) measured by flow cytometry following 48-hour co-culture of macrophages with ADC-pretreated 22Rv1.hSTEAP1 CM tumor cells. (E) Heatmap of MHC-I antigen presentation pathway gene expression from bulk RNA-seq of activated macrophages co-cultured with isotype, vandortuzumab, vandortuzumab–exatecan– or vandortuzumab–MMAE–treated tumor cells. (F) Heatmap of pro-inflammatory activation gene expression. (G) Schematic of the triple co-culture OT-I T-cell activation assay. 22Rv1.hSTEAP1.OVA cells were pretreated with ADCs, co-cultured with BMDMs for 48 hours to allow phagocytosis and antigen processing, then CellTrace Violet–labeled OT-I CD8^+^ T cells specific for SIINFEKL were added for 72 hours. (H–I) T-cell proliferation assessed by CellTrace Violet dilution (H) and frequency of CD44^+^ activated OT-I T cells (I) following triple co-culture. Data are represented as mean ± SD. Statistical comparisons by one-way ANOVA with Tukey’s multiple comparisons test. Dose–response curves were fit using a 4 PL regression model.

To nominate a lead candidate for in vivo evaluation in APMR models, we first assessed whether vandortuzumab-derived ADCs preserved the innate immune effector functions of the parental antibody in vitro. Additionally, we sought to determine if Fc functions of classical complement activation and Fcγ receptor engagement were important for ADC immune activation of myeloid cells. To this end, we engineered L234A, L235A, P329G (LALAPG) variants designed to abrogate both C1q binding and Fc engagement^42^. We first evaluated activation of the classical complement pathway by measuring murine complement deposition and CDC. We employed PC-3.hSTEAP1 and a bone metastasis–derived 22Rv1.hSTEAP1 model, termed caudal metastasis (CM), as the parental 22Rv1 line exhibited intrinsically high baseline complement deposition that confounded evaluation of ADC-mediated complement activation (Supplementary Fig. 4A). All WT ADC constructs (hereafter referred to as vandortuzumab) retained vandortuzumab’s intrinsic capacity to engage the classical complement pathway and murine complement deposition correlated with CDC activity (Fig. 4A, Supplementary Fig. 4A).

We next examined whether vandortuzumab-derived ADCs retained functional FcγR signaling capacity, given our prior findings in HER2 ADCs that Fc engagement, together with payload-induced immunogenic cell death, is required to drive macrophage activation and downstream CD8^+^ T-cell responses^15^. Again using JURKAT reporter assays for human FcγRIIIa and its murine homolog FcγRIV, we evaluated Fc signaling across our candidate ADCs and LALAPG-ADC variants. As expected, LALAPG constructs showed no detectable FcγR signaling. In contrast, vandortuzumab-ADC constructs retained FcγRIV signaling comparable to parental vandortuzumab and maintained higher FcγRIIIa activation than isotype and LALAPG-ADC controls (Fig. 4B, Supplementary Fig. 4B). To functionally validate these findings, we measured macrophage-mediated antibody-dependent cellular phagocytosis (ADCP) using pHrodo-labeled tumor cells co-cultured with CellTrace Violet–labeled murine bone marrow–derived macrophages. In both 22Rv1.hSTEAP1 CM and PC-3.hSTEAP1 cells, vandortuzumab ADCs elicited robust ADCP comparable to the parental antibody, whereas LALAPG ADCs showed baseline phagocytosis levels similar to the isotype control (Fig. 4C, Supplementary Fig. 4C).

### Fc Engagement and topoisomerase-I Payloads Cooperate to Enhance Myeloid Antigen Presentation

Given our previous observations that the combined impacts of payload class and Fc engagement macrophage activation and antigen presentation from our studies of T-DXd, we next co-cultured ADC-treated tumor cells with macrophages and assessed expression of MHC I, MHC II, and the co-stimulatory molecules CD40, CD80, and CD86 (Fig. 4D, Supplementary Fig. 4D–E)^15^. These assays revealed that both vandortuzumab-DXd and vandortuzumab-exatecan elicited markedly greater upregulation of antigen presentation and activation markers compared to vandortuzumab-MMAE. Moreover, this effect was dependent on intact Fc signaling, as LALAPG variants of the topoisomerase inhibitor ADCs exhibited substantially diminished macrophage activation. Notably, treatment with payload alone did not induce comparable upregulation of antigen presentation machinery, consistent with the necessity of FcγR signaling observed in our previous studies of T-DXd^15^.

To explore these myeloid immune stimulatory phenotypes at the transcriptional level, we next performed bulk RNA sequencing on macrophages following co-culture with treated tumor cells. Reactome pathway enrichment and fGSEA using the Hallmark collection both revealed that vandortuzumab–exatecan preferentially enriched immune effector programs, including antigen processing and presentation, Interferon Gamma and Alpha Responses, IL-6/JAK/STAT3 Signaling, and TNFα Signaling via NF-κB, which were consistent with a pro-inflammatory, antigen-presenting state, while vandortuzumab–MMAE enriched for proliferative and biosynthetic programs including E2F Targets and G2M Checkpoint, consistent with a less immunologically activated state (Supplementary Figs. 4F–G). To quantify these differences, composite gene set scores for macrophage inflammatory (Nos2, Il6, Ccl5, Tnf, and Cd86) and MHC class I antigen presentation (Tap1, Tap2, Psmb8, Psmb10, H2-D1, H2-K1, and B2m) signatures confirmed that vandortuzumab–exatecan drove significantly higher activation relative to vandortuzumab–MMAE, isotype control, and unconjugated vandortuzumab (Figs. 4E–F, Supplementary Fig. 4H). Analysis of complement-related transcripts further revealed upregulation of several complement components following vandortuzumab engagement relative to isotype control (Supplementary Fig. 4I), suggesting antibody-mediated complement activation may additionally contribute to macrophage priming. Collectively, these data demonstrate that maximal innate immune activation requires both immunogenic payload-induced tumor cell stress and functional Fc engagement.

### Topoisomerase ADCs Drives Greater T-cell Activation against Tumor Associated Antigens

To determine whether enhanced macrophage activation translated into functional T-cell stimulation, we employed a triple co-culture system. 22Rv1 CM cells engineered to express both STEAP1 and membrane-bound OVA (mOVA) were treated with ADCs in the presence of macrophages, followed by addition of SIINFEKL-specific OT-I CD8^+^ T cells (Fig. 4G). Consistent with our previous studies with T-DXd and T-DM1, vandortuzumab–DXd and vandortuzumab–exatecan induced greater T-cell activation, with higher frequencies of CD44^+^ and divided T cells compared with vandortuzumab–MMAE or payload-alone conditions (Figs. 4H–I, Supplementary Figs. 4J–K). Additionally, the Fc-silent LALAPG–DXd and LALAPG-exatecan constructs demonstrated significantly reduced T-cell stimulation, supporting the requirement for both intact FcγR engagement and phagocytosis by macrophages to present tumor antigens and elicit T cell stimulation. Together, these results demonstrate that ADC-induced innate immune activation facilitates activation of T cells against tumor-associated epitopes in vitro, which in combination may contribute to durable immune memory.

### Vandortuzumab-exatecan demonstrates substantial anti-tumor effects in immunodeficient prostate cancer models

**Figure 5:**
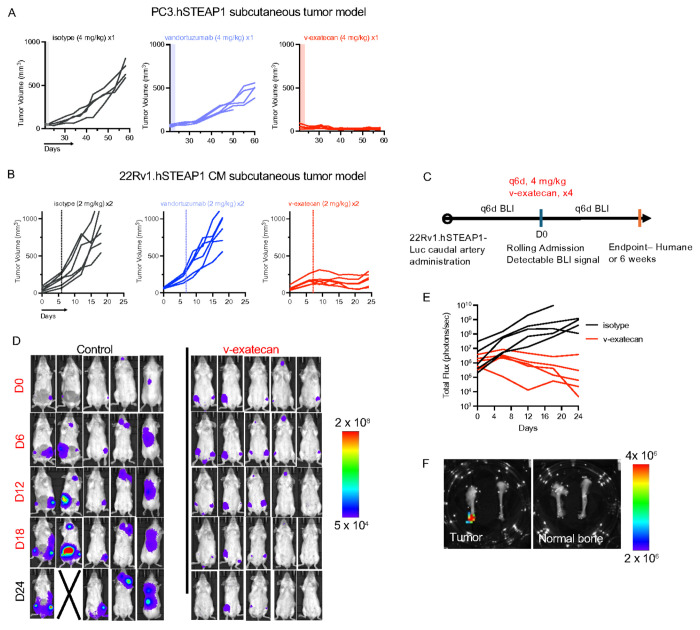
Vandortuzumab–exatecan demonstrates substantial antitumor activity in immunodeficient prostate cancer models. (A) Subcutaneous xenograft tumor growth curves in male SCID-Beige mice bearing established PC3.hSTEAP1 tumors treated with a single intraperitoneal dose of isotype control antibody, parental vandortuzumab, or vandortuzumab–exatecan (4 mg/kg). N ≥ 4–5 per group. (B) Therapeutic evaluation of vandortuzumab and vandortuzumab–exatecan in subcutaneous 22Rv1.hSTEAP1 caudal metastasis (CM) tumors in SCID-Beige mice. Animals received two intraperitoneal doses (2 mg/kg) administered three days apart. N=6 per group. (C) Schematic of tumor challenge experiments for 22Rv1.hSTEAP1-Luc CM disseminated models. Luc firefly luciferase, BLI bioluminescence imaging. (D) Representative bioluminescence images (IVIS) from SCID-Beige mice at indicated time points following caudal artery injection of 22Rv1.hSTEAP1-eGFP-Luciferase CM cells. Mice were randomized upon confirmed engraftment (7–14 days post-injection) and treated with isotype control or vandortuzumab–exatecan. N = 5 per group. (E) Quantification of total bioluminescence flux (photons/second) over time for individual mice in each treatment arm of the bone-metastatic model shown in (C). (F) Ex vivo bioluminescent imaging of representative femur and tibia demonstrates minimal background signal in controls and strong localization of signal to tumor-bearing site.

As an initial evaluation of in vivo antitumor activity in human prostate cancer models expressing STEAP1, we established subcutaneous PC3.hSTEAP1 xenografts in male SCID Beige mice. Once PC3.hSTEAP1 tumors reached approximately 100 mm^3^, animals received a single intraperitoneal dose (4 mg/kg) of isotype control antibody, parental antibody vandortuzumab, or vandortuzumab–exatecan. Treatment with vandortuzumab–exatecan resulted in significant and durable tumor growth inhibition through study endpoint (Fig. 5A, Supplementary Fig. 5A).

To model metastatic prostate cancer within the bone microenvironment, we utilized a caudal artery route of injection to establish and subculture a bone metastasis–derived 22Rv1.hSTEAP1.eGFP-Luciferase subline, as previously described (Supplementary Figs. 4A, 5B)^43^. Compared with the parental line, these CM cells exhibited a more homogeneous phenotype, with loss of a high-MFI CD44/EpCAM double-positive population observed in the parental line (Supplementary Fig. 5C). We first evaluated this line in a subcutaneous model, where two doses of 2 mg/kg vandortuzumab-exatecan was enough to induce a significant antitumor response relative to isotype control (Fig. 5B, Supplementary Fig. 5D). We next assessed therapeutic efficacy in a bone metastasis model. 22Rv1.hSTEAP1-luciferase CM cells were delivered via caudal arterial injection into SCID Beige mice (Fig. 5C), and tumor engraftment was monitored by bioluminescence imaging, with detectable signal emerging between 7 days and 2 weeks post-injection.

Mice were then randomized to receive four doses of 4 mg/kg isotype control antibody or vandortuzumab–exatecan, administered every six days. Animals receiving isotype control exhibited progressive disease, whereas vandortuzumab–exatecan treatment resulted in tumor stabilization or regression (Figs. 5D–F, Supplementary Fig. 5E). Importantly, these data demonstrate that vandortuzumab–exatecan is effective in the clinically relevant bone niche.

### Vandortuzumab-exatecan leads to adaptive anti-tumor immunity in a Syngeneic Prostate Cancer Model

**Figure 6.**
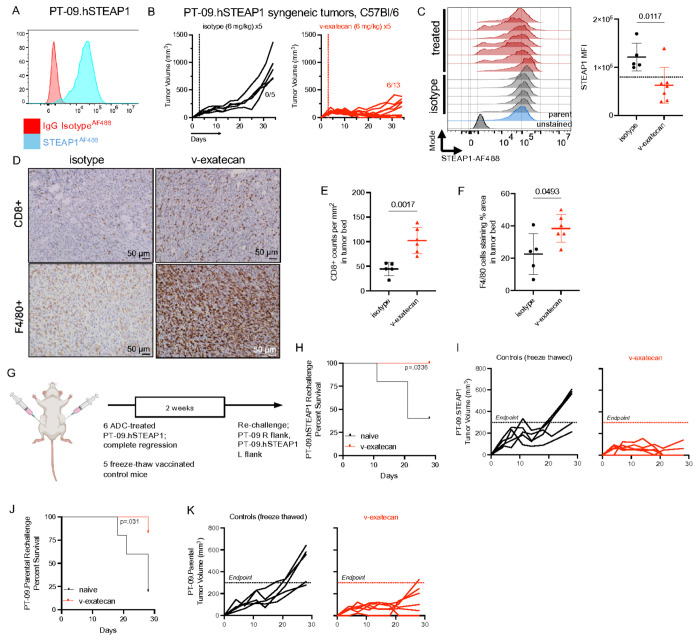
Vandortuzumab–exatecan induces durable adaptive anti-tumor immunity in a syngeneic prostate cancer model. (A) Expression of exogenously expressed hSTEAP1 in PT-09 cells via flow cytometry. (B) Tumor growth curves in C57BL/6 male mice bearing subcutaneous PT-09.hSTEAP1 tumors treated with vandortuzumab–exatecan (6 mg/kg, five doses every three days) or isotype antibody control. N=5 in isotype antibody control, N=13 in vandortuzumab-exatecan treated group. (C) STEAP1 surface expression on re-cultured residual PT-09.hSTEAP1 tumor cells from ADC-treated versus vehicle-treated mice and parent line measured by flow cytometry. (D) Representative IHC images of CD8^+^ T-cell (anti-CD8a antibody) and macrophage (anti-F4/80 antibody) infiltration in residual PT-09.hSTEAP1 tumor sections from vehicle- and vandortuzumab–exatecan–treated mice. Scale bars, 50 μm. (E-F) Quantification of CD8^+^ T-cell density (cells/mm^2^) and F4/80^+^ macrophage area (% positive area) in tumor sections from (D). (G) Schematic of the rechallenge experimental design. Complete responders (6 mice) were monitored for two weeks following treatment cessation, then rechallenged with bilateral subcutaneous implantation of PT-09.hSTEAP1 cells (right flank) and PT-09 parental cells lacking human STEAP1 (left flank). Freeze–thaw–inactivated PT-09.hSTEAP1 vaccinated naïve C57BL/6 mice served as controls. N=5 in freeze-thawed control mice. (H–I) Percent tumor-free survival (defined as tumor volume < 300 mm^3^) (H) and tumor growth kinetics (I) following PT-09.hSTEAP1 rechallenge. (J–K) Percent tumor-free survival (defined as tumor volume < 300 mm^3^) (J) and tumor growth kinetics (K) following rechallenge with PT-09 parental cells lacking human STEAP1. Data are represented as mean ± SD. Statistical comparisons by unpaired Welch’s t test. Survival/incidence by log-rank (Mantel–Cox) test.

Given the paucity of syngeneic prostate models in the field, we first advanced vandortuzumab–exatecan into the syngeneic EMT-6 model, a well-established murine epithelial tumor system frequently used to evaluate immune-mediated responses to ADCs (Supplementary Fig. 6A)^44,45^. In EMT-6.hSTEAP1 tumors, vandortuzumab–exatecan reduced tumor growth by 41% compared with vehicle control (mean tumor volume 267.1 mm^3^ vs. 649.5 mm^3^ at day 11; Supplementary Fig. 6B). Treatment was associated with increased infiltration of F4/80^+^CD11b^+^ macrophages and enhanced CD44 expression on tumor-infiltrating T cells relative to isotype controls (Supplementary Figs. 6B–C). Given the observed upregulation of CD40 during ADC-mediated antigen presentation and the importance of this axis in the induction of adaptive immunity, we next evaluated whether CD40 ligation could further potentiate ADC anti-tumor activity^46,47^. Combination treatment enhanced therapeutic efficacy beyond ADC or CD40 alone (mean tumor volume 1185 mm^3^ CD40 vs. 891 mm^3^ vandortuzumab-exatecan vs. 401 mm^3^ vandortuzumab-exatecan + CD40 at day 16; Supplementary Fig. 6D), supporting a functional role for CD40-dependent co-stimulation in amplifying ADC-induced immune responses. However, the rapid growth kinetics of EMT-6 tumors, relative insensitivity to vandortuzumab-exatecan, and the non–prostate origin of this system limited its utility for evaluating ADC adaptive immunomodulatory activity. We therefore sought to establish a murine prostate cancer model capable of responding to STEAP1-targeted ADC therapy, and thus we employed the PT-09 model. PT-09 cells are a syngeneic prostate cancer line derived from a tamoxifen-inducible Probasin-Cre;Pten^fl/fl^;Trp53^fl/fl^ genetically engineered mouse model on a C57BL/6 background that recapitulates the key genomic and androgen receptor signaling features of APMR^26^. Transduced PT-09.hSTEAP1 demonstrated sensitivity to both exatecan and vandortuzumab-exatecan in vitro (Fig. 6A, Supplementary Fig. 6E).

Using this model, we found that mice treated with 6 mg/kg twice weekly with vandortuzumab-exatecan demonstrated significant tumor regression compared to vehicle controls (mean tumor volume 118 mm^3^ vs. 925 mm^3^), with complete responses in 6 of 13 mice (46.2%) (Fig. 6B, Supplementary Fig. 6F). Residual tumors in both groups were excised, re-cultured, and analyzed for STEAP1 expression. ADC-treated tumors exhibited reduced STEAP1 expression (Fig. 6C), consistent with prior reports of target downregulation following STEAP1-directed therapy^11^. Immunohistochemical analysis of residual tumors revealed that vandortuzumab-exatecan treatment resulted in significantly increased infiltration of CD8 + T cells and F4/80 + macrophages infiltration into the tumor bed (Figs. 6D–F, Supplementary Fig. 6G). To evaluate whether these responses reflected the development of adaptive anti-tumor immunity, complete responders were rechallenged with PT-09.hSTEAP1 and PT-09 parental cells and compared with freeze-thawed vaccinated Bl/6 mice as summarized in Fig. 6G. Treated mice were protected from PT-09.hSTEAP1 rechallenge (Fig. 6H–I), with mean tumor volumes of 36 mm^3^ in treated mice versus 452 mm^3^ in freeze-thawed controls at 4 weeks (Supplementary Fig. 6F). Notably, treated mice also exhibited slower growth of PT-09 parental tumors lacking human STEAP1 (154.6 mm^3^ vs. 472 mm^3^ in control mice at 4 weeks), indicating the development of anti-tumor immunity not restricted to the STEAP1 antigen (Fig. 6J–K). Collectively, these results suggest that vandortuzumab-exatecan can elicit anti-tumor responses that are dependent upon its induction of durable anti-tumor immunity against PC.

## DISCUSSION

In this study, we examined the immune-mediated therapeutic properties of anti-STEAP1 ADCs by evaluating their ability to induce antigen presentation and T-cell epitope spreading in vitro and by developing an immunocompetent prostate adenocarcinoma model to assess immune contributions in vivo. Although prior preclinical studies of STEAP1-targeted ADCs demonstrated robust anti-tumor activity, these evaluations were conducted exclusively in immunocompromised models, limiting insight into immune-dependent mechanisms. Notably, vandortuzumab vedotin (vandortuzumab-VC-MMAE) showed strong efficacy in such models but failed to advance beyond phase I due to an unfavorable therapeutic index, underscoring the need for additional preclinical benchmarks that better predict clinical performance. Related to this point, our previous studies examined Trastuzumab-Deruxtecan (T-DXd) with Trastuzumab-Emtansine (T-DM1), which had demonstrated superior clinical efficacy in HER2 + breast cancer patients. In these studies, we found that T-DXd retained innate immune stimulatory properties of trastuzumab, which when combined with its payload, allowed for the promotion of antigen-presenting cell activation and subsequent T-cell responses, consistent with an in-situ vaccination effect that may contribute to durable tumor control. Importantly, this differential activity is driven by linker-payload design in combination with FcγR activity from the parent antibody. Applying similar immune-functional benchmarks in prostate cancer, in this study we compared vandortuzumab vedotin with vandortuzumab-VC-exatecan (payload modification) and vandortuzumab-GGFG-DXd (linker-payload modification). Across multiple immune activation metrics, we found that topoisomerase-based ADC constructs elicited superior induction of antigen presentation and T-cell activation relative to an MMAE-based ADC^48,49^.

We additionally examined the functional contribution of the Fc region in vitro, as several next-generation ADCs employ Fc-silent antibodies to minimize complement and Fcγ receptor–mediated effects^50,51^. While the unconjugated antibody lacked intrinsic anti-tumor activity in our prostate models, intact Fc signaling was required to drive optimal immune activation. ADCs lacking functional Fc domains showed reduced upregulation of antigen presentation markers and diminished downstream T-cell activation. Given that tumor-associated macrophages represent a dominant immune population in the prostate tumor microenvironment and serve as key antigen-presenting cells, these findings suggest that Fc engagement promotes phagocytosis and opsonization of dying tumor cells by macrophages, facilitating antigen processing and adaptive immune priming^52,53^. Taken together, these data indicate that ADC efficacy in prostate-specific immunocompetent settings depends not only on payload-mediated cytotoxicity but also on coordinated immune engagement mediated by both linker–payload design and Fc effector function.

Importantly, we extended evaluation of STEAP1-targeted ADCs into a site-specific bone metastasis model. Approximately 90% of men with advanced prostate cancer develop bone metastases, which are associated with substantial morbidity and mortality^54^. Despite this clinical reality, many preclinical ADC studies rely on subcutaneous xenografts that may not recapitulate the stromal and immune context of the bone microenvironment. In our study, vandortuzumab–exatecan retained anti-tumor activity within the bone metastatic niche, although higher dosing thresholds were required compared with subcutaneous models, suggesting that microenvironmental factors influence therapeutic efficacy. These findings underscore the importance of evaluating ADCs within clinically relevant metastatic sites, particularly in diseases such as prostate cancer where bone involvement predominates.

To interrogate immune-dependent mechanisms in vivo, we developed a syngeneic PTEN/TP53-deficient prostate tumor subcutaneous model (PT-09) in immunocompetent hosts. Immunocompetent modeling of advanced prostate cancer remains limited due to the scarcity of murine lines that recapitulate key genomic drivers of APMR and the intrinsic insensitivity of many murine tumors to ADC-mediated cytotoxicity. Such baseline resistance can limit tumor cell stress and antigen release, obscuring downstream immune effects. In contrast, PT-09 retained measurable sensitivity to STEAP1-targeted ADCs, enabling interrogation of both cytotoxic and immune-mediated mechanisms. Consistent with prior reports of STEAP1-directed therapies, ADC treatment led to downregulation of STEAP1 expression, suggesting that target modulation may represent a mechanism of resistance and could contribute to the limited durability observed clinically with earlier STEAP1-directed ADCs such as vandortuzumab–vedotin. Notably, vandortuzumab–exatecan induced durable tumor regression and protected mice from rechallenge with antigen-negative tumor cells, supporting the development of adaptive immune responses not restricted to the STEAP1 antigen. Together, these findings suggest that effective ADC platforms must engage immune mechanisms capable of controlling tumors even after target downregulation, potentially through macrophage-mediated antigen presentation and epitope spreading.

One limitation of this study is the absence of endogenous STEAP1-expressing APMR xenograft models in vivo. However, both C4-2B and 22Rv1 demonstrated complement deposition that was independent of classical pathway activation, complicating interpretation of antibody-mediated effects. We therefore utilized engineered or derived cell lines that lacked this baseline complement activity to enable more accurate assessment of ADC-mediated complement responses. Another limitation of our study is the absence of a direct in vivo comparison between vandortuzumab–MMAE and vandortuzumab–exatecan in immunocompetent models. Such comparisons require dose normalization across constructs with distinct cytotoxic potency. Because Fc engagement and immune activation exhibit threshold-dependent behavior, differential dosing could confound interpretation of immune-mediated effects, particularly in systems where modest changes in Fcγ receptor signaling produce measurable differences in macrophage activation and T-cell priming. We therefore prioritized immune-functional screening assays—including macrophage antigen presentation and T-cell activation readouts in tumor models with comparable IC_50_ values—to guide candidate selection for in vivo testing. This strategy enabled isolation of immune-functional differences independent of overt cytotoxic potency and supports the premise that immune activation may represent a more clinically relevant benchmark than maximal in vitro cytotoxicity alone. Future studies could address this limitation by optimizing drug-to-antibody ratios or dosing strategies to achieve comparable cytotoxic thresholds in immunocompetent prostate models and evaluating immune memory responses under matched potency conditions.

Collectively, our findings suggest that ADC efficacy in prostate cancer depends on coordinated interactions among payload-induced tumor cell stress, Fc-mediated innate immune engagement, and adaptive immune priming. Certain linker–payload architectures may therefore convert ADCs from purely cytotoxic agents into immunologic amplifiers capable of inducing durable anti-tumor responses. Incorporating immunocompetent and site-specific metastatic models into preclinical evaluation may provide more predictive insight into clinical performance and help distinguish constructs with similar cytotoxic potency but divergent immune activation potential.

## Supplementary Material

This is a list of supplementary files associated with this preprint. Click to download.

• SupplementaryFigures.pdf

## Data Availability

The datasets generated and analyzed during the current study, including raw RNA sequencing data, will be deposited in the NCBI Gene Expression Omnibus (GEO) prior to publication and will be publicly accessible at that time. Previously generated datasets reanalyzed in this study are available in publicly accessible repositories. TCGA Prostate Adenocarcinoma (PRAD) RNA-seq data and corresponding normal prostate tissue data from the Genotype-Tissue Expression (GTEx) project were accessed through the UCSC Xena Browser [https://xenabrowser.net/datapages/?cohort=TCGA+TARGET+GTEx]. Metastatic castration-resistant prostate cancer transcriptomic data from the SU2C/PCF Dream Team cohort were accessed through the cBioPortal for Cancer Genomics [https://www.cbioportal.org/study/summary?id=prad_su2c_2019] and are additionally available through dbGaP under accession number phs000915.v2.p2 [https://www.ncbi.nlm.nih.gov/projects/gap/cgi-bin/study.cgi?study_id=phs000915.v2.p2]. Custom code used for pixelwise H-score quantification of IHC images in QuPath and analysis of bulk RNASeq is available on GitHub [https://github.com/johnswang1/STEAP1_ADC_scripts] and archived on Zenodo [10.5281/zenodo.19099957].
